# Molecular identification and antimicrobial activities of some wild Egyptian mushrooms: *Bjerkandera adusta* as a promising source of bioactive antimicrobial phenolic compounds

**DOI:** 10.1186/s43141-021-00200-8

**Published:** 2021-07-19

**Authors:** Elham R. S. Soliman, Heba El-Sayed

**Affiliations:** Botany and Microbiology Department, Faculty of Science, Helwan University, Helwan, Egypt

**Keywords:** Phylogenetic, HPLC, Fruiting fungi, rRNA-ITS

## Abstract

**Background:**

The discovery of potential, new cost-effective drug resources in the form of bioactive compounds from mushrooms is one way to control the resistant pathogens. In the present research, the fruiting bodies of five wild mushrooms were collected from Egypt and identified using internal transcribed spacer region (ITS) of the rRNA encoding gene and their phylogenetic relationships, antimicrobial activities, and biochemical and phenolic compounds were evaluated.

**Results:**

The sequences revealed identity to *Bjerkandera adusta*, *Cyclocybe cylindracea*, *Agrocybe aegerita*, *Chlorophyllum molybdites*, and *Lentinus squarrosulus* in which *Cyclocybe cylindracea* and *Agrocybe aegerita* were closely related, while *Chlorophyllum molybdites* was far distant. *Cyclocybe cylindracea* and *Agrocybe aegerita* showed 100% similarity based on the sequenced ITS-rDNA fragment and dissimilar antimicrobial activities and chemical composition were detected. *Bjerkandera adusta* and *Cyclocybe cylindracea* showed strong antimicrobial activity against *Escherichia coli*, *Pseudomonas aeruginosa*, *Staphylococcus aureus*, *Micrococcus luteus*, *Streptococcus pneumoniae*, and *Candida albicans*. This activity could be attributed to the detected phenolic and related compounds’ contents.

**Conclusion:**

Our finding provides a quick and robust implement for mushroom identification that would facilitate mushroom domestication and characterization for human benefit.

## Background

Mushrooms belong to a special group of macroscopic fungi with distinctive fruiting bodies of Basidiomycota and some belong to Ascomycota [1]. In areas with high humidity, wild mushrooms grow naturally on tree trunks or rotting woody debris. They are used as nutritious foods and therapeutic sources worldwide [2, 3], as well as excellent recyclers and decomposers [4], and thus play a significant role throughout the environment. They are typically abundant worldwide during the wet season and have been discovered grown on different substrates [5, 6].

Mushrooms have a high content of proteins, carbohydrates, crude fibers, minerals, vitamins, and secondary metabolites, as well as a source of bioactive substances that promote good health, e.g., antioxidants, antimicrobials, anticancer, cholesterol-lowering, and immune-stimulating effects have been reported for certain mushroom species [7–9]. Accurate identification knowledge of edible mushrooms is essential for effective exploration in human benefits. About 140,000 different species of mushrooms exist on the planet; only about 10% is well characterized. As macroscopic fungi, mushrooms are identified depending on their morphological characters. Nowadays, molecular markers are prevailed in the identification of different organisms because of their robustness and efficiency. Identification based on direct sequencing of the internal transcribed spacers region of the rRNA encoding gene is a precise tool for identification and constructing phylogenetic relationships among different organisms or species [10]. Nineteen mushroom samples of *Termitomyces aurantiacus*, *Tricholoma matsutake*, *Tricholoma robustum*, *P*. *ostreatus*, *Schizophyllum commune*, and *Pleurotus pulmonarius* collected from Southwest Nigeria were successfully identified using the rDNA-ITS region and the obtained sequences were used to construct their phylogenetic relatedness with reference strains in the NCBI gene-bank [11]. Molecular identification using the rDNA-ITS region as a basis for species identification was generated for 33 representative mushroom samples collected from powdered mycelium samples, grocery store mushrooms, and capsules from commercial dietary supplements. Generally, the ITS sequences were efficaciously applied to verify and certify dietary supplements containing mushrooms [12].

Extracts from several mushroom species have been reported to have antimicrobial activity derived from their phenolic contents [13–15]. Phenolic acids prevailed in mushrooms, including benzoic and cinnamic acid derivatives. In numerous mushroom species, p-hydroxybenzoic, protocatechuic, gallic, vanillic, and syringic acids have been found. Cinnamic acid and its derivatives, such as P-coumaric acid, O-coumaric acid, caffeic acid, ferulic acid, and chlorogenic acid, were also reported. Certain flavonoids such as quercetin, rutin, and chrysin have been identified [16].

The present study aimed to employ molecular characterization for the identification of morphologically different mushrooms collected from Egypt. The discovery of new cost-effective drug resources in the form of bioactive compounds from mushrooms as a promising source of them is one way to control drug-resistant pathogens. In this regard, the bioactivity of mushroom extracts was evaluated in an attempt to explore new natural sources for controlling widespread microbes affecting human health.

## Methods

### Mushroom samples

**Fig. 1:**
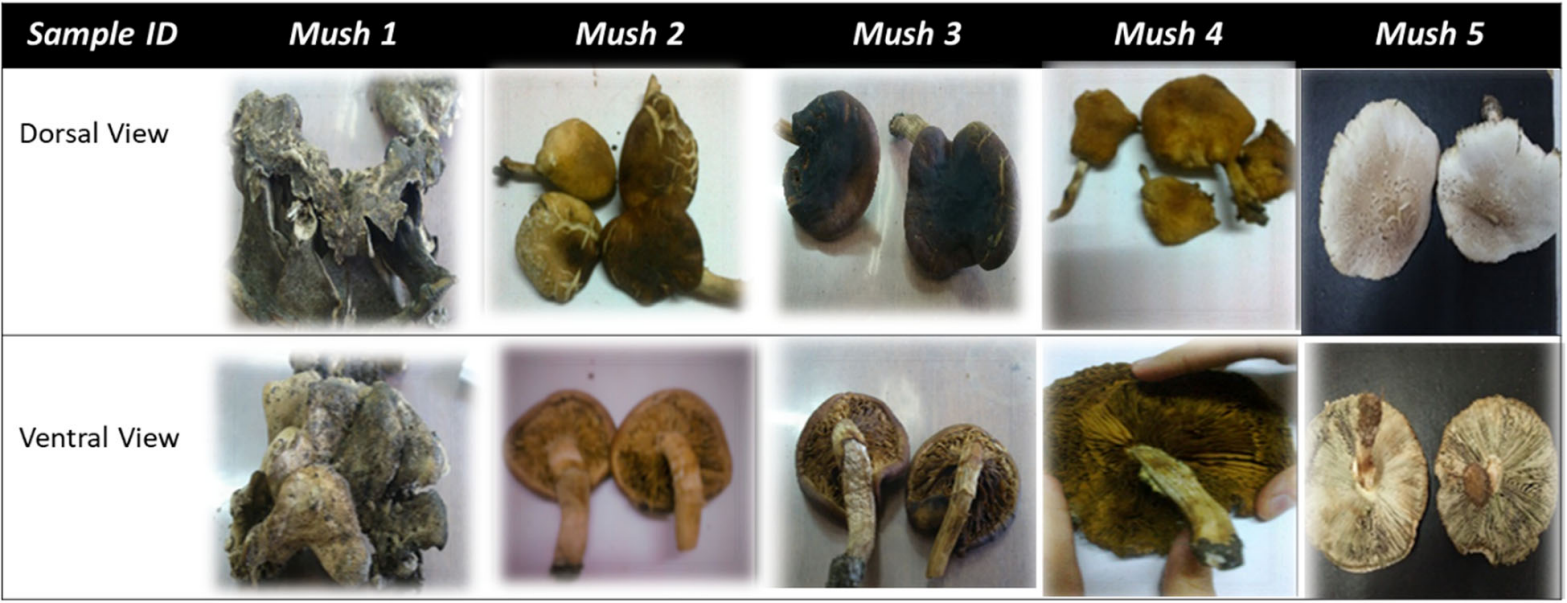
Morphological characterization of the collected wild mushroom fruiting bodies

Five morphologically different wild mushrooms labeled *Mush* 1 to 5 were collected from their natural habitat in Al-Beheira Governorate and Cairo, Egypt, between January and February 2017. The mushroom samples were characterized morphologically following the methodology suggested by Largent and Stuntz [17]. The samples verified up to genus level [18] as follows: *Mush* 1, *Bjerkandera sp*.; *Mush* 2 and 3, *Cyclocybe sp*.; *Mush* 4, *Chlorophyllum* sp.; and *Mush* 5, *Lentinus* (Fig. 1). The samples were dried at room temperature to a constant weight, then preserved in a dry place until use. The macroscopic features of the collected mushroom were recorded. The specimens collected were deposited in Mycology Laboratory, Faculty of Science, Helwan University, Egypt.

### DNA isolation and molecular identification

Total genomic DNA was isolated from fruit bodies according to [19]. The total isolated DNA were column purified using DNA Purification MiniSpin Kit (VIOGENE cat# PF1001) according to the manufacturer’s protocol. The purified DNA was used as a template for PCR reaction using Mytaq Red DNA polymerase master mix (BIOLINE cat # BIO-21108) according to manufacture instructions. Briefly, the reaction was containing 1× PCR red master mix buffer, 2.0 μl of 10 pm/μl of each primer; ITS1 (5′ TCCGTAGGTGAACCTGCGG 3′) and ITS4 (5′ TCCTCCGCTTATTGATATGC 3′), 1.0 μl of DNA template (~ 30 ng), 0.25 μl of MyTaq™ DNA polymerase (5 U/μl), then the total volume was adjusted to 50 μl using sterile water. The amplification reactions were performed in Thermal Cycler (Biometra, Germany) as follow: 1st cycle of 3 min at 95 °C for initial denaturation, followed by 35 cycles of 20 s at 95 °C (denaturation), 20 s at 55 °C (annealing), 30 s at 72 °C (extension), and then a final extension was carried out for 10 min at 72 °C; the reaction was held at 4 °C. The PCR products were separated on 1% agarose gel, the amplified fragments were purified using a PCR-M clean-up system (VIOGENE cat# PF1001) according to the manufacturer’s protocol, followed by sequencing with ITS1 primer at GATC Company using ABI 3730xl DNA-sequencer.

### Sequencing data analysis

The obtained nucleotide sequences were aligned to the total nucleotide collection of NCBI using Basic Local Alignment Search Tool for nucleotide blast (https://blast.ncbi.nlm.nih.gov/Blast.cgi). The phylogenetic tree is constructed using the UPGMA tree build method with 100 bootstrapping in Geneious 8.1.9 software [20].

### Preparation of ethanol crude extracts

Dried fruiting bodies were grounded in a grinder into a fine powder before extraction. Five grams of the powdered samples were extracted with 95% ethanol (50 mL) at 30 °C at constant shaking of 150 rpm for 24 h. The extracts were centrifuged at 3000 rpm, 4 °C for 15 min to give a clear supernatant that was filtered through Whatman filter paper number 4. The clear filtrate was concentrated by evaporation under vacuum, then the crude extract was stored at 4 °C for further use [21]. The crude extracts have been evaluated for their antimicrobial activity.

### Test microorganisms and growth conditions

The antimicrobial activity of extracts from the investigated mushrooms was tested against 6 different pathogenic bacterial strains and one yeast species, namely, *Candida albicans ATCC1031*. *Escherichia coli ATCC25922*, *Pseudomonas aeruginosa ATCC 7853*, and *Staphylococcus aureus ATCC25923* were kindly provided from Pharmaceutical Control Authority, Giza, Egypt, while *Proteus mirabilis*, *Micrococcus luteus*, and *Streptococcus pneumoniae* were in-house isolates. The strains were routinely cultured in Nutrient agar (Diffco) medium at 37 °C. A single colony from each corresponding organism was inoculated into Nutrient broth medium and allowed to grow for 24 h at 37 °C. The grown cultures were used for the antimicrobial assay.

### *In vitro* antimicrobial activity assay of mushroom’ extracts

Antimicrobial assay of mushroom extracts was verified using the well diffusion method [22]. The inoculum size of each test organism was adjusted to a concentration of 1.5 × 10^8^ CFU/mL by comparing with 0.5 McFarland standards. Bacteria and yeast seeded plates were prepared by inoculating 100 μl suspension of each tested culture into nutrient agar media. Wells were made on the agar surface with a 6-mm cork borer. The extracts were dissolved in sterile distilled water to a final concentration of 10 μg/ml. A hundred microliters of extracts were poured independently into the well using a sterile syringe. The seeded plates were refrigerated for 8 h at 4 °C to allow extract diffusion into agar then, were incubated at 37 ± 2 °C for 24 h. The plates were observed for the inhibition zone formation around the wells.

The inhibition zone was calculated by measuring the diameter of the inhibition zone around the well (in millimeters) including the well diameter in three independent replicas. The measurements were taken in three different fixed directions for each plate and the average values for the three independent replicas were tabulated. Gentamycin (10 μg/disk) was used as a standard reference antibiotic.

### Chemical composition of the crude extract of *Cyclocybe cylindracea* and *Agrocybe aegerita* fruiting bodies

Even identified differentially, *Mush* 2 and 3 show 100% similarity based on the sequenced ITS-rDNA fragment that argues for the same species for both samples; however, dissimilar antimicrobial activities were detected. Therefore, both samples were used to evaluate their chemical compositions by measuring the total soluble sugars, proteins, and phenolic contents.

#### Total soluble sugars

As described by Umbreit et al., total sugars were estimated using the anthrone technique [23]. Six milliliters anthrone solution (2 g anthrone /L H_2_SO_4_ of 95 %) was added to 3 ml mushroom extract and the mixture was kept in a boiling water bath for 3 min. The formed color was measured at 620 nm by a spectrophotometer after cooling. The calibration curve was constructed with different concentrations of glucose as a standard. Total soluble sugar content of the sample was determined as glucose equivalent and expressed as milligrams per gram of the extract.

#### Total soluble proteins

Total soluble proteins were determined according to Lowry et al., [24]. One milliliter of each mushroom extract was mixed with 5 ml of freshly prepared solution of 2% sodium carbonate, 4% sodium hydroxide, and 0.5% copper sulfate in 1% sodium tartrate. The mixture was incubated at room temperature for 10 min before the addition of 0.5 ml Folin and made up to 10 ml. After 30 min, the optical density of the mixture was measured at 750 nm. The calibration curve was constructed with different concentrations of albumin bovine serum as a standard. The total soluble proteins were calculated using albumin bovine serum equivalent and expressed as milligrams per gram of the extract.

#### Total phenolic contents

Using Folin-Ciocalteu reagent and gallic acid as a standard, total phenolic contents were determined according to Kujala et al., (2000) [25]. Half a millimeter of each mushroom’s extract was mixed well with 2.5 mL of 1:10 ethanol diluted Folin-Ciocalteu’s reagent, 2 mL of 7.5% Na_2_CO_3_. After 15 min incubation at room temperature, the absorbance of mixtures was recorded by spectrophotometer at 765 nm. Different concentrations of gallic acid as standard have been prepared to construct the calibration curve. The total phenolic contents were calculated as gallic acid equivalent (GAE) and expressed as milligram per gram of the extract (mg GAE/g sample extract).

### HPLC analysis

According to Roberts et al., (2018) [26] HPLC was carried out at the National Research center, Cairo, Egypt using Agilent 1260 series with a C18 column (4.6 mm × 250 mm i.d., 5 μm) at 35 °C to identify and quantify the phenolics and related compounds present in *Bjerkandera adusta* extract as it displayed the highest antimicrobial activity [26]. The mobile phase consisted of water (A) and acetonitrile (B) at a flow rate of 1 ml/min. The multi-wavelength detector was monitored at 280 nm. The sample injection volume was 10 μl. For the quantitative analysis of phenolic compounds, a calibration curve was obtained by injection of different concentration of gallic acid, chlorogenic acid, catechin, caffeine, syringic acid, rutin, ellagic acid, coumaric acid, vanillin, ferulic acid, naringenin, propyl gallate, 4′,7-dihydroxyisoflavone, quercetin, and cinnamic acid standards.

### Statistical analysis

All analyses were performed in triplicate. The data were recorded as means ± SD (standard deviation). The statistical (SPSS version 17.0) package program was used to perform one way ANOVA. P ≤ 0.05 is considered statistically significant.

## Results

### Molecular identification of mushroom samples

**Fig. 2:**
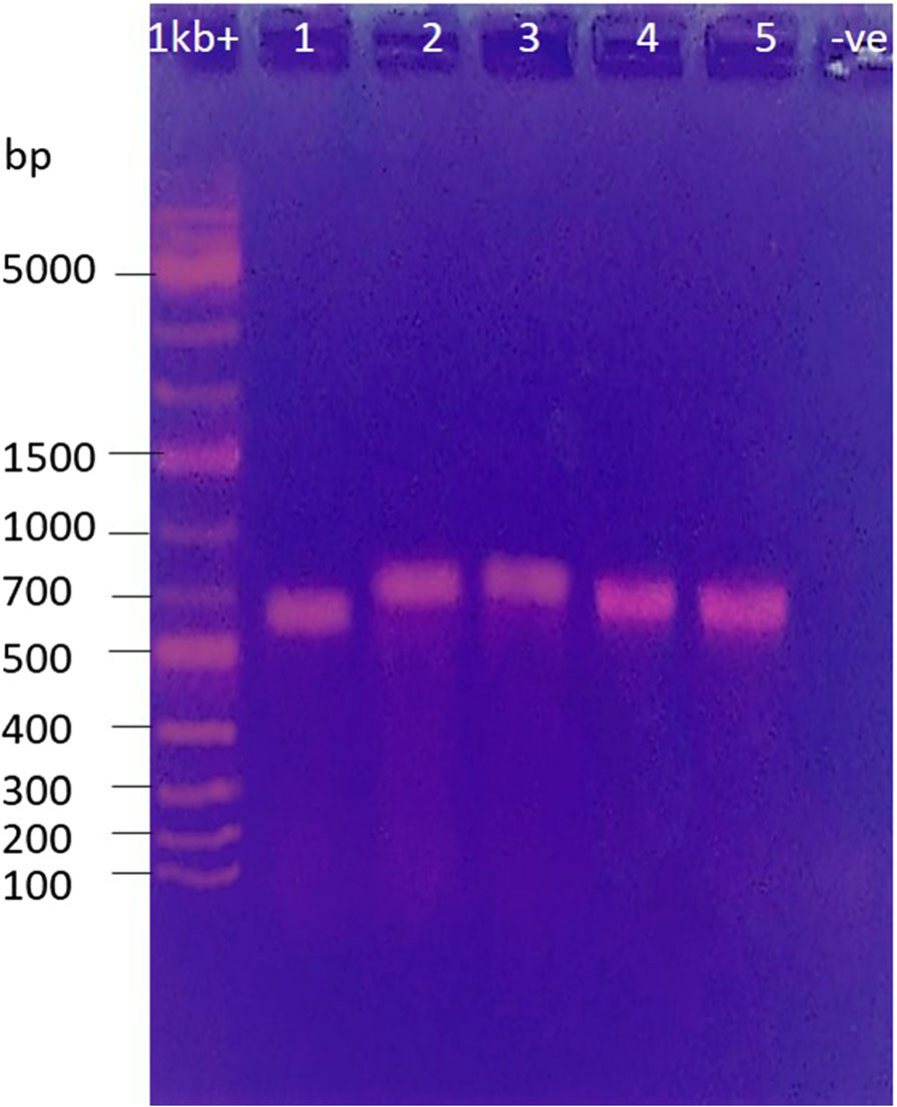
PCR products amplified from five mushroom samples; lanes 1 to 5, 1 Kb^+^ refers to the DNA ladder; –ve refers to negative control of the PCR; no band indicates no PCR contamination

The amplified fragments of the ITS-rRNA encoding gene were varied in length among the collected samples. A similar fragment length of about ~ 900 bp was amplified from *Mush* 2, 3, and 4 while a fragment length of ~ 700 bp was amplified from *Mush* 1 and a fragment length of ~ 800,bp was amplified from *Mush* 5 (Fig. 2).

The nucleotide sequences were aligned against the whole nucleotide collection database using the Basic Local Alignment Search Tool (BLAST) search program of the National Center for Biotechnology Information (NCBI) site. All the sequences were deposited into the NCBI database and the acquired accession numbers are listed in Table 1 with the BLAST results.

**Table 1:** Summary of the NCBI nucleotide BLAST results and the deposited sequences’ accession IDs

Samples ID	Sequence coverage %/identity %	Identified organism (ID)	Accession ID for the deposited sequences
*Mush* **1**	100/99.8	*Bjerkandera adusta* (KX426963)	MN602043
*Mush* **2**	95/88.8	*Cyclocybe cylindracea* (KM260145)	MN646676
*Mush* **3**	97/89.2	*Agrocybe aegerita* (AY763674)	MN635615
*Mush* **4**	81/93.4	*Chlorophyllum molybdites* (MG270072)	MN638772
*Mush*** 5**	100/96.7	*Lentinus squarrosulus* (KF150321)	MN602044

Based on percent of identity, mushrooms may be identified as follows: *Mush* 1 shows 99.8% identity to *Bjerkandera adusta* (accession KX426963). Both *Mush* 2 and 3 show 88.8 and 89.2% identity to both *Cyclocybe cylindracea* voucher TO AV97345a (accession KM260145) and *Agrocybe aegerita* strain SM970201 (accession AY763674), respectively. *Mush* 4 shows 93.3% identity to *Chlorophyllum molybdites* strain LS091 (accession MG270072). *Mush* 5 shows 96.7% identity to *Lentinus squarrosulus* (accession KF150321).

**Fig. 3:**
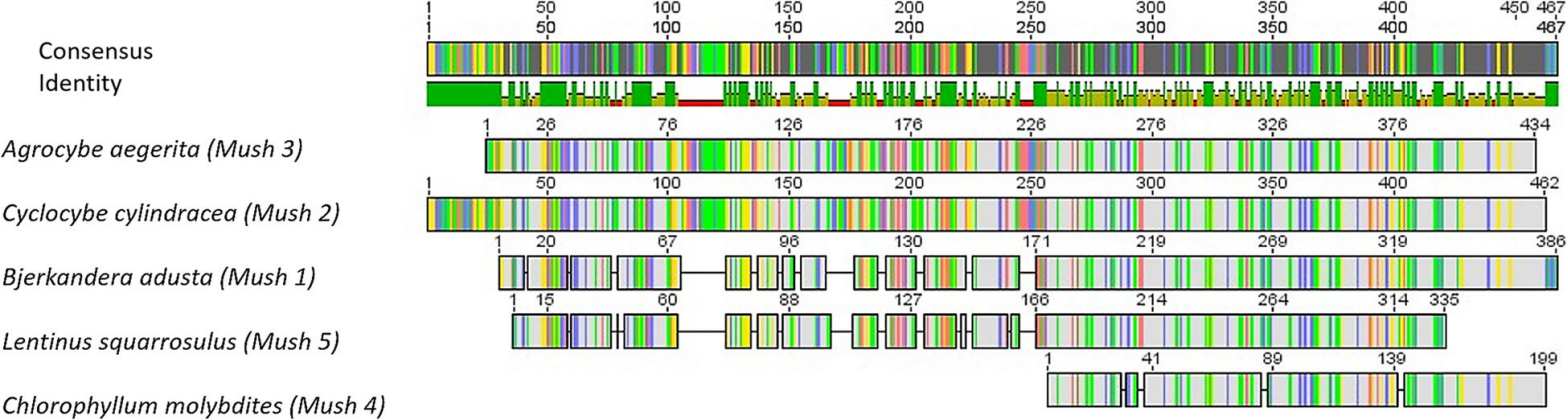
Nucleotide alignment graph showing the pairwise similarities between the five mushrooms; *Bjerkandera adusta*, *Cyclocybe cylindracea*, *Agrocybe aegerita*, *Chlorophyllum molybdites*, and *Lentinus squarrosulus* based on ITS sequencing data

All ITS sequences were aligned to each other to determine the pairwise similarities between sequences using quantity one software. The results show a maximum similarity of 100% was detected between *Cyclocybe cylindracea* and *Agrocybe aegerita* while the lowest similarity was detected between *Chlorophyllum molybdites* and all other mushrooms, Table 2 and Fig. 3.

**Table 2:** Percentage of pairwise similarity between five mushrooms; *Bjerkandera adusta*, *Cyclocybe cylindracea*, *Agrocybe aegerita*, *Chlorophyllum molybdites*, and *Lentinus squarrosulus* based on ITS sequencing data

	*Bjerkandera* (*Mush 1*)	*Cyclocybe* (*Mush 2*)	*Agrocybe* (*Mush 3*)	*Chlorophyllum* (*Mush 4*)	*Lentinus* (*Mush 5*)
*Bjerkandera* (*Mush *1)					
*Cyclocybe* (*Mush* 2)	58.89				
*Agrocybe* (*Mush* 3)	59.21	100			
*Chlorophyllum* (*Mush* 4)	42.65	35.44	35.64		
*Lentinus* (*Mush* 5)	82.06	58.03	58.03	45.45

**Fig. 4:**
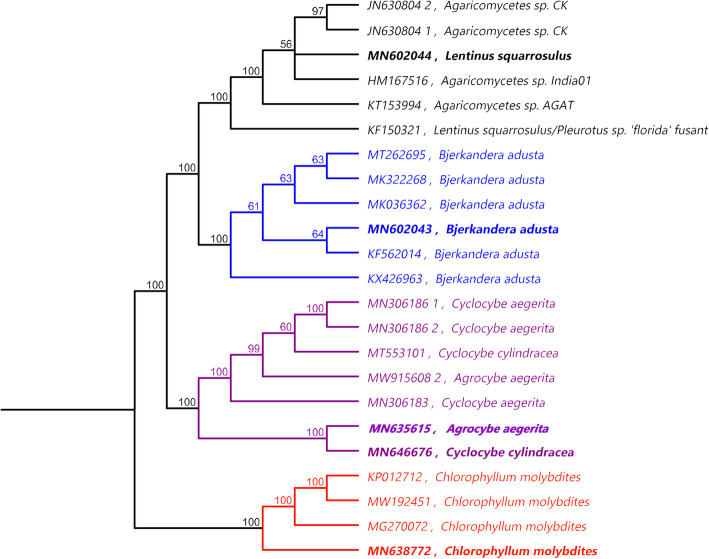
phylogenetic tree generated using UPGMA tree build method based on ITS sequencing data of the different five mushroom samples (bold font) in relation to the most closest accessions in the database. The numbers on the nodes indicating the percent of 100 bootstrapping

The phylogenetic tree constructed revealed three major clades. The first clade includes the closely related species, *Bjerkandera adusta* and *Lentinus squarrosulus*. The second clade includes *Cyclocybe cylindracea* and *Agrocybe aegerita* with identical phylogenetic relationships. On the other hand, *Chlorophyllum molybdites* showed a distant relationship with other samples and was separated in the third clade, Fig. 4.

### *In vitro* antimicrobial activity

**Fig. 5:**
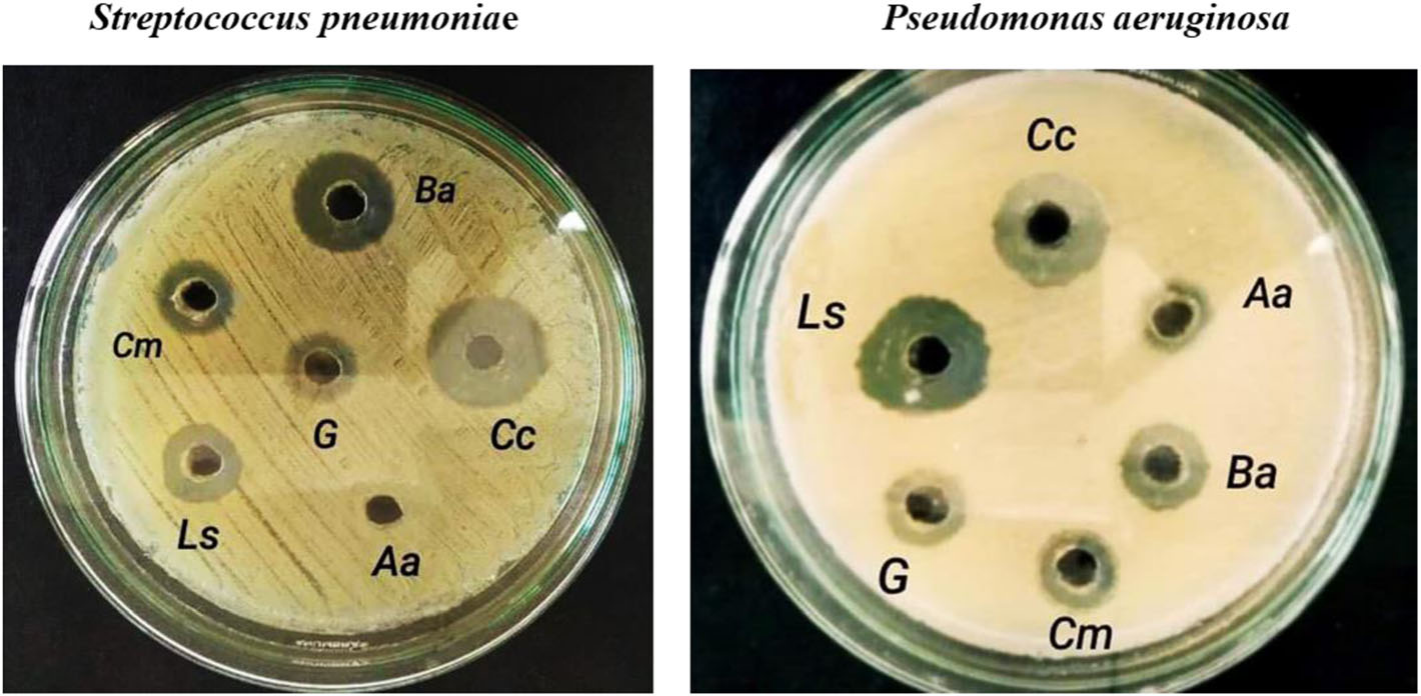
Petri plates showing antimicrobial assay of different fungal ethanolic extracts against *Streptococcus pneumoniae* and *Pseudomonas aeruginosa* using well diffusion method. Letters on the plates refer to the fungal species: Ba, *Bjerkandera adusta*; *Cc*, *Cyclocybe cylindracea*; *Aa*, *Agrocybe aegerita*; *Cm*, *Chlorophyllum molybdites*; *Ls*, *Lentinus squarrosulus*; and G, Gentamycin 10 μg/well

Six pathogenic Gram-positive and Gram-negative bacteria and one yeast were screened against ethanol extracts from the studied mushroom strains to verify their antimicrobial activities. Except for *Proteus mirabilis*, the ethanolic extracts of *Bjerkandera adusta* and *Cyclocybe cylindracea* had the most effective antimicrobial activity against all the microorganisms examined. The largest inhibition zone of 22.0 ± 0.10 mm was detected for *Bjerkandera adusta* extract against *Micrococcus luteus* while its lowest inhibition zone (10.0 ± 0.52mm) was detected against *Pseudomonas aeruginosa ATCC7853*. *Agrocybe aegerita* showed only very weak antimicrobial activity against *Pseudomonas aeruginosa ATCC 7853* and *Escherichia coli ATCC25922* of 2 mm inhibition zone. Ethanolic extracts of *Chlorophyllum molybdites* and *Lentinus squarrosulus* demonstrated strong antimicrobial activity against *Streptococcus pneumoniae*, *Pseudomonas aeruginosa ATCC 7853*, and no activity against the other strains. Gram-positive bacteria were more sensitive to the ethanolic extracts of *Bjerkandera adusta* and *Cyclocybe cylindracea* than Gram-negative bacteria (Table 3 and Fig. 5).

**Table 3:** Antimicrobial activities for the ethanolic extracts of different wild mushroom strains by using a well diffusion method

Pathogenic microbial strains		The diameter of inhibition zone (mm)*					
		Gentamycin (10 μg/well)	***Bjerkandera adusta*** (***MN602043***)	***Cyclocybe cylindracea*** (***MN646676***)	***Agrocybe aegerita*** (***MN635615***)	***Chlorophyllum molybdites*** (***MN638772***)	***Lentinus squarrosulus*** (***MN602044***)
**Gram-positive bacteria**	***Streptococcus pneumonia*e**	10.0 ± 0.00	17.0 ± 0.20	20.0 ± 0.29	-	12.0 ± 0.33	15.0 ± 0.10
	***Staphylococcus aureus ATCC25923***	8.0 ± 0.00	15.0 ± 0.10	13.0 ± 0.12	-	-	-
	***Micrococcus luteus***	9.0 ± 0.00	22.0 ± 0.10	14.0 ± 0.22	-	-	-
**Gram-negative bacteria**	***Escherichia coli ATCC25922***	2.0 ± 0.00	12.0 ± 0.30	17.0 ± 0.20	2.0 ± 0.00	-	-
	***Pseudomonas aeruginosa ATCC 7853***	10.0 ± 0.00	10.0 ± 0.52	17.0 ± 0.00	2.0 ± 0.00	10.0 ± 0.41	20.0 ± 0.22
	***Proteus mirabilis***	-	-	-	-	-	-
**Yeast**	***Candida albicans ATCC1031***	-	12.0 ± 011	14.0 ± 0.00	-	-	-

Values for the zone of growth inhibition (measured as the diameter of the clear zone around the well) are means ± SD of three independent replicas. The diameter of the well (5 mm) is included

“-” indicates no inhibition

### Chemical composition of the crude extract of *Cyclocybe cylindracea* and *Agrocybe aegerita* fruiting bodies

**Fig. 6:**
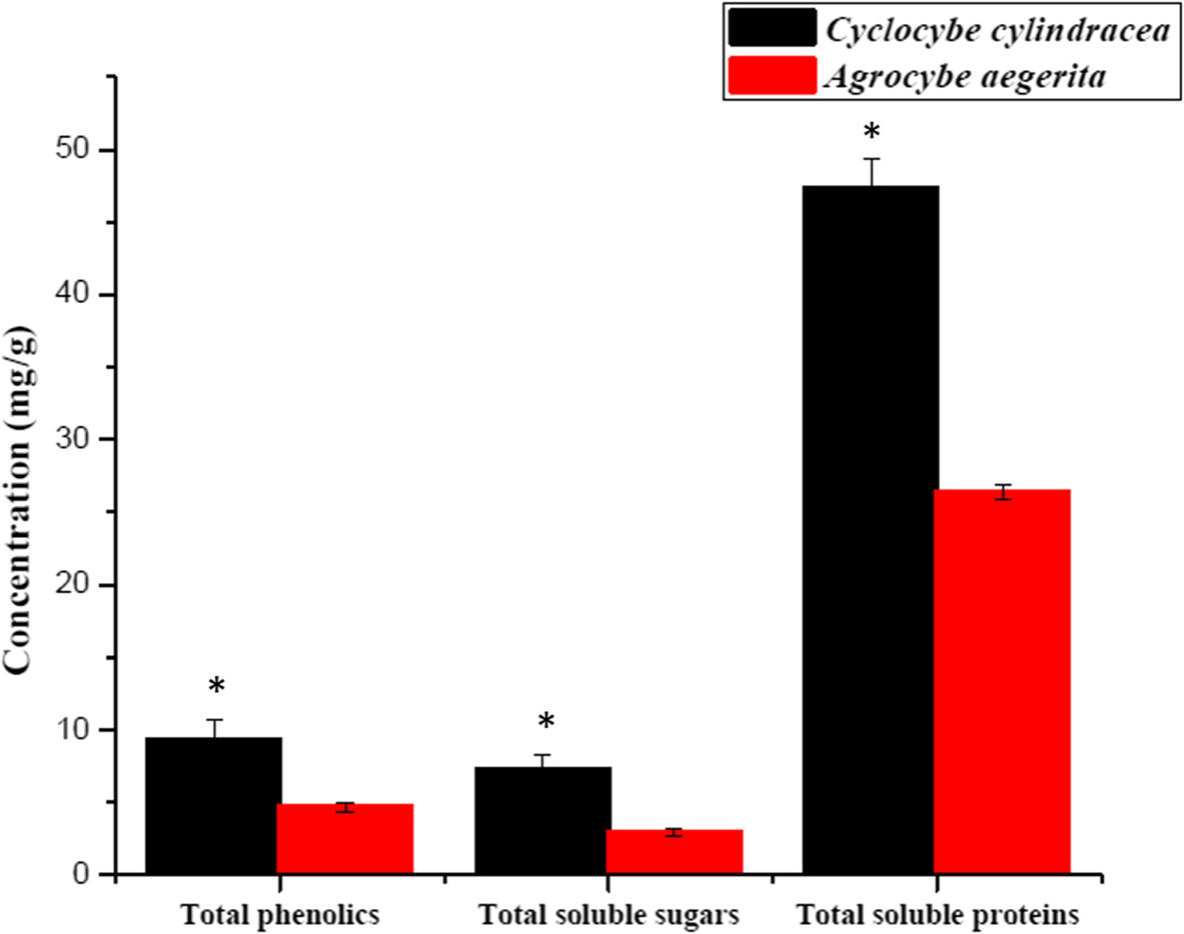
Chemical composition of the crude extract of *Cyclocybe cylindracea* and *Agrocybe aegerita* fruiting bodies. Asterisk refers to significance at p < 0.05

*Cyclocybe cylindracea* and *Agrocybe aegerita* extracts were chemically analyzed for their different antimicrobial activity while they show 100% pairwise similarity for the ITS amplified fragment. The total phenols, sugars, and soluble protein content were varied between both samples. Interestingly, the highest contents were observed for *Cyclocybe cylindracea*. The data were represented in Fig. 6.

### HPLC analysis of *Bjerkandera adusta* ethanolic extract

The highest antimicrobial activity was observed for *Bjerkandera adusta* ethanolic extract. Therefore, we aimed to identify its chemical constituents using HPLC. Many phenolics and related compounds (gallic acid, chlorogenic acid, catechin, caffeine, coffeic acid, syringic acid, rutin, propyl gallate, 4′,7-dihydroxyisoflavone, quercetin, and cinnamic acid) were positively identified and quantified in the *Bjerkandera adusta* ethanolic extract as compared with the chromatographic characteristics and absorption spectra of standard compounds. Chlorogenic acid was the highest concentration of 11.33 μg/ml while only 0.47 μg/ml of gallic acid was the least, see Table 4.

**Table 4:** Bioactive phenolic compounds detected in the *Bjerkandera adusta* ethanolic extract using HPLC analysis

Compound name	Formula	Retention time (min)	Molecular mass (g mol^**−1**^)	Peak area	Area %	Conc. (μg/ml)
**Gallic acid**	C_7_H_6_O_5_	3.07	170.12	9.17	0.17	0.47
**Chlorogenic acid**	C_16_H_18_O_9_	3.45	354.31	204.81	3.74	11.33
**Catechin**	C_15_H_14_O_6_	3.83	290.26	25.88	0.47	5.88
**Caffeine**	C_8_H_10_N_4_O_2_	4.06	194.19	84.02	1.54	2.10
**Coffeic acid**	C_9_H_8_O_4_	4.82	180.16	62.04	1.13	1.63
**Syringic acid**	C_9_H_10_O_5_	5.15	198.17	38.75	0.71	1.24
**Rutin**	C_27_H_30_O_16_	5.55	610.517	34.17	0.62	3.93
**Propyl gallate**	C_10_H_12_O_5_	10.29	212.2	88.14	1.61	2.74
**4′,7-dihydroxyisoflavone**	C_15_H_10_O_4_	10.44	254.241	101.21	1.85	1.86
**Quercetin**	C_15_H_10_O_7_	10.57	302.236	79.52	1.45	4.46
**Cinnamic acid**	C_9_H_8_O_2_	11.16	C9H8O2	855.07	15.62	5.37

## Discussion

With the growing demand for edible and medicinal mushrooms, research into the rich biodiversity of fungi is significant. Not only is it important to classify such high-quality fungal species, but it has significant economic importance, as it can help to detect new bioactive products. Agaricomycetes serve as degrading organisms, pathogens, parasites, and ectomycorrhizal symbionts of forest trees. Mushroom identification using the ITS region has been proven to be efficient in taxonomic identification. ITS-based identification is specific and rapid, requires only a small amount of sample, and can apply for dried samples [12]. However, this approach does not directly reflect the dietary supplement contents or pharmacological activity of the specimen being studied [27]. In the present study, the ITS region of the rRNA encoding gene was successfully used to identify five wild mushrooms collected from two localities in Egypt: Al-Beheira Governorate and Cairo. Homology between sequences identified from samples was used to construct the phylogenetic relationship among the characterized species. The ITS region sequences of the *Mush* 1 matched the BLAST reference sample for *Bjerkandera adusta*. Interestingly, both *Mush* 2 and 3 show high sequences similarities to both *Cyclocybe cylindracea* and *Agrocybe aegerita*, respectively. Furthermore, the ITS nucleotide similarity and phylogenetic tree show that the *Cyclocybe cylindracea* is genetically identical to *Agrocybe aegerita*. The only acceptable explanation for the current observation is that both samples are representing one species. Based on DNA sequence analysis, Vizzini et al., (2014) [28] stated that *Cyclocybe cylindracea* is the synonymous new name to *Agrocybe aegerita* [28]. Nevertheless, both samples displayed differential antimicrobial activities that could be associated with different isolates as ecological habitats greatly influence chemical composition and physiological pathways for better fitness with the surrounding environment. Otherwise, it may be attributed to different stages of development and different chemical composition found in extracts [11]. In agreement, total phenolics, sugars, and proteins were higher in *Cyclocybe cylindracea* in comparison to *Agrocybe aegerita* (Fig. 6). Until recently, Niveiro and his coauthor were described the *Cyclocybe cylindracea* and *Agrocybe aegerita* as two independent genera which could explain the varied antimicrobial activities and the varied chemical composition of both samples [29].

ITS region sequences of *Mush* 4 matched the *Chlorophyllum molybdites* while *Mush* 5 shows homology to *Lentinus squarrosulus*. For all characterized samples, the morphological features were consistent with the molecular data up to species levels. The majority of the edible mushrooms are Agaricomycetes and being collected from the garden [30]. In agreement, the ITS-based identification of mushrooms collected for the present study shows that all belong to Agaricomycetes [31]. In the current study, most of the ITS sequences of different mushrooms native to Egypt are not 100% homology with the reference gene sequence in NCBI GenBank, which may reflect the impact of the ecological habitat where the mushrooms are grown [30].

Our result validates how conserved sequences of mushrooms are primarily principle for their molecular identification and extended to understand their evolutionary basis. Detailed phylogenetic analyses showed that all mushroom samples fall into three major clades; *Cyclocybe cylindracea* and *Agrocybe aegerita* were genetically identical and separated together in one clade while *Lentinus squarrosulus* show ~82% pairwise similarity to *Bjerkandera adusta* and so both of them are represented in the second clade. *Chlorophyllum molybdites* show the least pairwise similarity to all other mushrooms in which it reached a maximum of ~42% with *Bjerkandera adusta* and a minimum of ~35% with both *Cyclocybe cylindracea* and *Agrocybe aegerita*. Therefore, it was phylogenetically distinct from all others and was separated in the third clade. In accordance with Binder et al., (2005) [32] *Bjerkandera adusta* and *Lentinus squarrosulus* was phylogenetically close and grouped in a polyporoid clade while *Chlorophyllum molybdites* was phylogenetically distinct and distributed in the euagaricus clade [32].

The molecular taxonomy of wild mushrooms would enable researchers and people interested in the mushroom industry to detect the current widespread existing species and the endangered species to protect [12]. This direction would be applicable if the molecular identification of mushrooms is coupled with the identification of their antimicrobial activities or their chemical constituents to evaluate their economic importance whether as a source of food or as pharmaceutical products.

Recently, new effective metabolites against pathogenic microorganisms resistant to traditional treatments should be established. Here, as an alternative, wild mushrooms were evaluated. In pursuit of new antimicrobial agents, mushrooms were screened for antimicrobial activity [33]. Different types of mushrooms were found to display different antimicrobial behaviors. In the present study, ethanolic extracts from five wild mushrooms have been evaluated for antimicrobial activity against various pathogens. Screening of the antimicrobial activity against Gram-positive, Gram-negative bacteria, and the yeast of the ethanolic wild mushroom extracts showed that *Agrocybe aegerita*, *Chlorophyllum molybdites*, and *Lentinus squarrosulus* had little activity against pathogenic bacteria and yeast examined. Alternatively, except for *Proteus mirabilis*, *Bjerkandera adusta* and *Cyclocybe cylindracea* extracts exhibit relatively strong antimicrobial activity against all bacteria and yeast tested. For extracts, the inhibition region exhibiting more than 10 mm was considered highly active. Barneche et al., (2016) [34] reported that *Bjerkandera adusta* exhibited antimicrobial activity against *Xanthomonas vesicatoria*, *Aspergillus oryzae*, *Penicillium expansum*, *Botrytis cinerea*, and *Rhizopus stolonifera* [34]. In contrast to the current result, Chikwem et al., (2020) observed that *B*. *adusta* aqueous, methanol, and ethanol extracts demonstrated no antimicrobial activity against *Candida albicans*. However, some *B*. *adusta* extracts were highly effective against three Gram-negative bacteria (*Escherichia coli*, *Salmonella typhimurium*, and *Pseudomonas aeruginosa*) and three Gram-positive bacteria (*Staphylococcus aureus*, *Staphylococcus epidermidis*, and *Bacillus cereus*) [35]. In agreement with Kumar et al., (2016) [36] *Cyclocybe cylindracea* displayed a strong antimicrobial activity against Gram-positive *Staphylococcus aureus* and Gram-negative *Klebsiella pneumonia* and *E*. *coli* that was attributed to its polysaccharide content [36]. Overall, the strength of the antimicrobial activity depends on the mushroom species, chemical composition, and the microbe being tested.

Phytochemicals, especially phenolic compounds and flavonoids, have been considered as a valuable source of relatively cheap and safe antimicrobial agents with very few adverse effects [37]. There have been limited investigations concerning the individual profiles of phenolic compounds in wild mushrooms. The phenolic content of the ethanol extract of the most potent species, *Bjerkandera adusta*, was investigated in our study. HPLC analysis revealed 11 different phenolic compounds with various concentrations. Previous studies have confirmed the presence of various derivatives of benzoic acid, cinnamic acid, and its derivatives, and some flavonoids in various mushroom species [15, 38]. Chlorogenic acid was the highest phenolic detected of 11.33 μg/ml, the presence of it may explain the strong antimicrobial activity of *Bjerkandera adusta* as it bounds the pathogen’s outer membrane disrupting its permeability, defect barrier activity, causing minor leakage of nucleotide and cytoplasmic material consequently cell death [39]. Catechin was also detected to 5.88 μg/ml. Catechin produces hydrogen peroxide that damages bacterial cell membranes [40]. Gallic acid of 0.47 μg/ml was quantified in our sample in which the antimicrobial activity could be attributed to it. Gallic acid exhibits antimicrobial action against different bacteria, such as *Pseudomonas* strains [41]. Additionally, it improves antimicrobials efficiency possibly by inhibiting efflux pumps, which are important mechanism for producing antimicrobial resistance [42, 43]. 1.63 μg/ml of caffeic acid was quantified in *Bjerkandera adusta* extract, it associates with cell membrane integrity destruction and interferes with *S*. *aureus* cell aerobic metabolism [44]. Numerous studies have shown that flavonoid antibacterial mechanisms (quercetin-flavonol, 4′,7-dihydroxyisoflavone, rutin) mainly include inhibition of nucleic acid synthesis, inhibition of cytoplasmic membrane function by affecting the formation of biofilms, porins, permeability, and interaction with certain critical enzymes [45, 46]. Overall, the observed strong antimicrobial activity of *Bjerkandera adusta* could be attributed to synergistic interactions between various natural phenolics and flavonoids detected in its extract.

The results from this research are of particular importance in terms of finding new, wild species of medicinal mushrooms that contain bioactive components. The antimicrobial potential proven in the analyzed mushroom extracts shows that they can be used in the food industry as a substitute for synthetic antimicrobial compounds. At the same time, the extract can be a basis for use in alternative medicine.

## Conclusion

In conclusion, an accurate phylogenetic analysis establishes a theoretical method for defining a classified status of new edible or medicinal mushrooms. Besides, their evolutionary relationships could provide an important clue for further exploration of the active compounds. Furthermore, molecular characterization is the authentication of wild mushrooms. Drug-resistant microbial strains have been creating serious treatment problems. This situation has forced the search for new antimicrobial drugs effective against pathogenic microorganisms. Wild mushrooms, as a natural source, could be an alternative being included in the diet. The mechanism of antibacterial action of the best extracts (e.g., *Bjerkandera adusta* and *Cyclocybe cylindracea*) should be elucidated and the individual/combined compounds found in those extracts might be tested against selected bacteria to identify molecules responsible for the bioactivity.

## Data Availability

All data generated or analyzed during this study are included in this article.

## Abbreviations

rDNA-ITS: Ribosomal DNA internal transcribed spacers regions
Mush: Mushroom
HPLC: High-performance liquid chromatography
NCBI: National Center for Biotechnology Information
BLAST: Basic Local Alignment Search Tool
